# Cerebrospinal Fluid Cutaneous Fistula Following Neuraxial Anesthesia in a Placenta Previa Case: A Rare Complication

**DOI:** 10.7759/cureus.96511

**Published:** 2025-11-10

**Authors:** André Silva, Rita Oliveira, Raquel Pontes, Catarina Filipe, Ana Milheiro

**Affiliations:** 1 Anesthesiology and Critical Care, Unidade Local de Saúde Gaia/Espinho, Vila Nova de Gaia, PRT

**Keywords:** cerebrospinal fluid cutaneous fistula, cesarian sections, combined spinal-epidural anaesthesia, intrathecal complication, placenta previa

## Abstract

Cerebrospinal fluid (CSF) cutaneous fistula is a rare complication of neuraxial anesthesia, caused by CSF leakage through the epidural puncture site, usually after catheter removal. It may present with symptoms of intracranial hypotension, but some cases remain asymptomatic, making diagnosis and management less straightforward. Evidence is scarce, mostly from case reports, and no standardized treatment exists. Our case emphasizes that early recognition and a stepwise, minimally invasive approach can effectively manage cerebrospinal fluid fistulas, even in asymptomatic obstetric patients.

We present a case report of a 36-year-old nulliparous woman at 37 weeks with placenta previa who underwent elective cesarean section under combined spinal-epidural anesthesia (CSE) at L4-L5. The procedure was uneventful, and postoperative pain was well controlled. On postoperative Day 2, after epidural catheter removal, a clear fluid leak was observed at the puncture site, increasing with the Valsalva maneuver. The patient remained apyretic and asymptomatic, with no headache, photophobia, or neurological signs.

Given the classical cesarean section (vertical hysterotomy), increased intra-abdominal pressure was suspected to contribute to persistent leakage. A multidisciplinary discussion led to initial conservative management (bed rest, hydration, pressure dressing). As the leak persisted after 24 hours, a simple cutaneous suture was performed, resulting in immediate closure. She was discharged on day five and remained asymptomatic at four-week follow-up.

This case illustrates that CSF fistula may occur even after uncomplicated neuraxial anesthesia and remain clinically silent. High suspicion is essential, as bedside tests and beta-2 transferrin have limitations. Conservative measures are usually effective, but a simple suture is a safe, minimally invasive option when leakage persists. Multidisciplinary collaboration optimizes management, especially in obstetric patients.

## Introduction

In obstetric anesthesia, neuraxial techniques are considered the gold standard for both cesarean delivery and labor analgesia [1]. The combined spinal-epidural (CSE) technique is frequently chosen because it allows for quick onset of anesthesia while maintaining pain relief through an epidural catheter [2]. Although CSE is widely used and generally safe, rare complications such as cerebrospinal fluid (CSF) cutaneous fistula can occur.

A CSF cutaneous fistula is characterized by abnormal leakage of cerebrospinal fluid from the subarachnoid space through the epidural puncture site, typically after the removal of the epidural catheter. This condition is uncommon, with a reported incidence of approximately 0.16% following neuraxial procedures, and may arise from spontaneous, traumatic, or iatrogenic causes [3]. Potential symptoms include intracranial hypotension with postural headaches, nausea, vomiting, photophobia, and phonophobia, and in severe cases, it may lead to complications such as meningitis or subdural hematoma [4]. Prompt recognition and treatment are essential to prevent these serious outcomes. 

Only a few cases have been described in the literature, most following either accidental dural puncture or spinal surgery, and reports in obstetric patients remain particularly scarce [5]. Despite the potential risks, CSF fistulas after neuraxial anesthesia are not well-documented in the literature, and there is no standardized treatment protocol.

In this report, we present the case of a 36-year-old woman, 37 weeks of gestation, who developed a CSF cutaneous fistula after an uneventful CSE anesthesia for an elective cesarean section because of a placenta previa.

## Case presentation

A 36-year-old nulliparous woman with a height of 158cm and a BMI of 26 kg/m2, with no previous medical history, presented for an elective cesarean section at 37 weeks’ gestation due to placenta previa. The anesthetic plan involved CSE anesthesia using a “needle-through-needle” technique, with an 18-G Tuohy needle and a 27-G Whitacre pencil-point spinal needle from a sequential

The combined spinal-epidural block was performed using the sequential “needle-through-needle” technique at the L4-L5 interspace, with the patient in the sitting position. The epidural space was identified on the first attempt without technical difficulty, using the loss-of-resistance to saline technique at a depth of 4 cm. A 27-G Whitacre spinal needle was then introduced through an 18-G Tuohy needle to perform the subarachnoid puncture, and 8 mg of hyperbaric bupivacaine combined with 2 µg of sufentanil were administered intrathecally. The epidural catheter was subsequently threaded to a depth of 8 cm at the skin level, to provide the option for intraoperative supplementation and postoperative analgesia, and correct placement was confirmed with a negative aspiration test and initial bolus effect.

**Figure 1:**
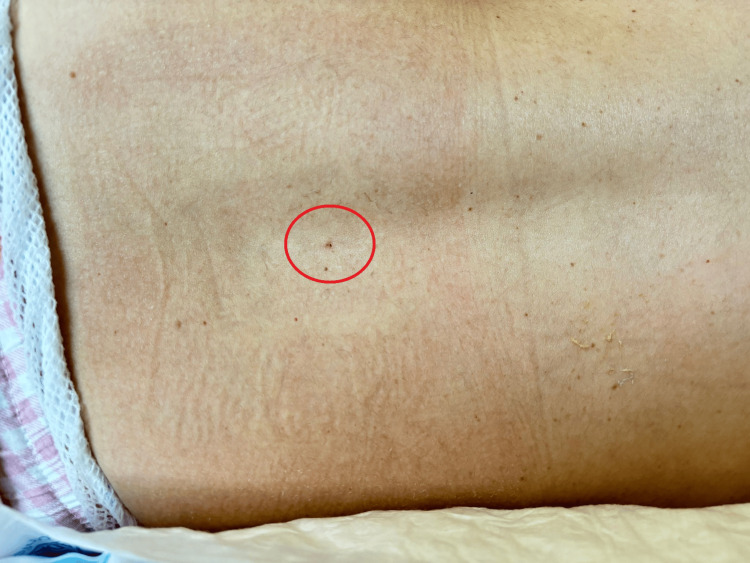
Epidural puncture site Epidural catheter removed on the second postoperative day, with moderate amount of fluid observed draining from the epidural insertion site.

Due to the location of the placenta, the surgical team performed a classical cesarean section with a vertical uterine incision rather than the more common transverse approach. Postoperative pain was managed effectively through intermittent epidural boluses of 0.2% ropivacaine, supplemented by intravenous paracetamol and ketorolac. Pain scores recorded at 24 hours post-surgery were 0 at rest and 3 with movement. The epidural catheter was removed on the second postoperative day, and a small amount of clear fluid was observed at the insertion site (Figure 1). 

On examination, the fluid appeared to leak spontaneously from the puncture site, with a slight increase during Valsalva maneuvers. The lumbar region showed no swelling. A bedside urine test strip was used on the leaking fluid, showing a positive result for glucose, supporting the suspicion of cerebrospinal fluid leakage. Neurological assessment revealed intact motor and sensory function, and both Kernig’s and Brudzinski’s signs were negative, with no evidence of meningeal irritation. Vital signs remained stable, and the patient was afebrile. Apart from mild nausea, intracranial hypotension was deemed unlikely as she did not experience any headache, vomiting, photophobia, back pain, neck stiffness, seizures, or fever.

After consultation with neurosurgery, a conservative approach was initially adopted, consisting of hydration, strict bed rest, and application of a compressive dressing. The patient showed a gradual decrease in CSF leak without complications. However, when the CSF leak persisted the following day, a direct closure of the puncture site was performed, immediately resolving the leak. She was discharged on postoperative day five. At the four-week follow-up, the patient remained asymptomatic and reported no sequelae.

## Discussion

Reports on CSF cutaneous fistulas following neuraxial anesthesia are sparse, and the exact mechanisms leading to this complication remain poorly defined. Several mechanisms have been proposed to explain its formation, including unintentional dural puncture during needle or catheter insertion, particularly after multiple attempts; persistence of a dural defect or micro-tear after puncture; delayed tissue healing due to infection, steroid use, or poor nutritional status; and increased intra-abdominal or epidural pressure, as may occur during pregnancy, obesity, or classical cesarean section. Additionally, removal of the epidural catheter may create a tract that facilitates CSF leakage through the puncture site [6]. In this case, our patient underwent a combined spinal-epidural block for a classical cesarean section due to placenta previa, a procedure that involves a vertical hysterotomy. This technique increases intra-abdominal pressure compared to the low transverse incision, potentially contributing to the formation of a CSF cutaneous fistula.

Diagnosing a CSF cutaneous fistula can be challenging due to the absence of standardized laboratory or imaging protocols. Bedside methods, such as multiparameter test strips (Combur-Test®), may provide initial guidance but are limited by low sensitivity and specificity, which can lead to false results. Beta-2 transferrin remains the gold standard for confirming the presence of CSF [7]. In our case, a bedside urine reagent strip was used on the leaking fluid, which tested positive for glucose, supporting the suspicion of a CSF leak; however, beta-2 transferrin testing was not performed due to limited availability, cost constraints, and sufficient clinical confidence in the diagnosis based on the patient’s presentation and evolution.

There is no universally accepted treatment algorithm for CSF cutaneous fistulas. Different management options have been reported, ranging from conservative to surgical approaches. Table 1 summarizes the main strategies described in the literature.

**Table 1:** Reported management strategies for CSF cutaneous fistula after neuraxial anesthesia Content adapted from references [5,8,9] CSF: cerebrospinal fluid

Approach	Strategy	Typical Outcome
Conservative	Bed rest, hydration, pressure dressing, analgesia	Most cases resolve within a few days without invasive measures
Minimally invasive	Cutaneous suture at puncture site	Rapid closure in persistent leaks; simple and effective
Epidural blood patch	Injection of autologous blood near leak	High success rate but contraindicated in anticoagulated patients
Surgical repair	Direct dural closure (rare)	Reserved for refractory or extensive leaks

Conservative management is typically the first-line approach, focusing on measures to reduce CSF loss and relieve symptoms of intracranial hypotension, including bed rest, adequate hydration, and analgesia. When conservative strategies fail, more direct interventions may be considered, such as cutaneous suturing of the puncture site or an epidural blood patch. Suturing creates a local tamponade effect that can effectively seal the leak, while an epidural blood patch forms a clot to close the fistula; however, the latter may be contraindicated in patients receiving anticoagulants [7]. 

In this case, the patient remained largely asymptomatic, consistent with reports indicating that significant fluid leaks can occur without intracranial hypotension or other clinical manifestations [10]. Initially, a conservative approach was attempted, but persistent leakage ultimately necessitated closure with a cutaneous suture, resulting in complete resolution [6,11]. An epidural blood patch was not performed due to the absence of clinical symptoms and the decision to avoid an unnecessary invasive procedure in a hemodynamically stable postpartum patient, as the potential risks outweighed the expected benefits in this context, although our patient had a normal coagulation profile.

Obstetric patients present additional considerations when managing such complications. Minimizing disruption to postpartum care and supporting maternal-infant bonding, including breastfeeding, are important. In this patient, early discharge enabled uninterrupted newborn care by the parents, and proximity to the hospital allowed for prompt access to medical services if required.

## Conclusions

In summary, CSF cutaneous fistula after neuraxial anesthesia is a rare but important complication, and current evidence remains limited to case reports. Maintaining a high index of suspicion is essential for early recognition, even in asymptomatic patients, particularly when predisposing factors such as increased intra-abdominal pressure are present.

Most cases respond well to conservative management - bed rest, hydration, pressure dressings, and analgesia - but when leakage persists, simple surgical closure can offer a safe and effective solution. Although serious complications such as meningitis or subdural hematoma are uncommon, prompt identification and a multidisciplinary approach remain key to ensuring optimal outcomes in obstetric patients.
